# Preliminary clinical experience of robot-assisted surgery in treatment with genioplasty

**DOI:** 10.1038/s41598-021-85889-w

**Published:** 2021-03-18

**Authors:** Li Lin, Cheng Xu, Yunyong Shi, Chaozheng Zhou, Ming Zhu, Gang Chai, Le Xie

**Affiliations:** 1 Institute of Forming Technology & Equipment, Shanghai Jiao Tong University, Xuhui Campus, 1954 Hua Shan Rd, Shanghai, 200030 China; 2 Department of Plastic and Reconstructive Surgery, Shanghai 9Th People’s Hospital, School of Medicine, Shanghai Jiao Tong University, 639 Zhi Zao Ju Rd, Shanghai, 200011 China; 3 Institute of Medical Robotics, Shanghai Jiao Tong University, Minhang Campus, 800 Dong Chuan Rd, Shanghai, 200240 China; 4 The College of Medical Instrument, Shanghai University of Medicine & Health Sciences, No. 257, Zhouzhu Highway, Pudong Campus, Shanghai, 200120 China; 5 Department of Plastic and Reconstructive Surgery, Maternal and Child Health Care Hospital of Hainan Province, Haikou, 570206 China; 6 National Digital Manufacturing Technology Center, Shanghai Jiao Tong University, Xuhui Campus, 1954 Hua Shan Rd, Shanghai, 200030 China

**Keywords:** Translational research, Biomedical engineering

## Abstract

Genioplasty is the main way to treat diseases such as chin asymmetry, dysplasia and overdevelopment, which involve the three-dimensional direction abnormalities of the chin. Since this kind of surgery mainly uses intraoral incisions, the narrow surgical field of intraoral incisions and the surrounding important neurovascular tissues make it easy for complications, to occur during the osteotomy process, which results in greater surgical risks. The first craniofacial-plastic surgical robot (CPSR-I) system is developed to complete the precise positioning and improve the surgeon's force perception ability. The Kalman filtering method is adopted to reduce the interference of sensor signal noise. An adaptive fuzzy control system, which has strong robustness and adaptability to the environment, is designed to improve the stability of robot-assisted surgical operations. To solve the problem of the depth perception, we propose an automatic bone drilling control strategy that combines position and force conditions to ensure that the robot can automatically stop when the bone is penetrated. On the basis of model surgery and animal experiments, preliminary experiments were carried out clinically. Based on the early results of 6 patients, the robot-assisted approach appears to be a safe and effective strategy for genioplasty.

## Introduction

Genioplasty is a common procedure in craniofacial surgery. It is mainly used to repair facial bone tissue to restore the normal shape, function or appearance. These patients can have chin asymmetry, bone warts, and chin retraction, among other clinical manifestations. Some syndromes are also accompanied by functional defects, which seriously affect the quality of life of patients. Thus, the procedure makes the facial contour more symmetrical and enhances the confidence of patients in social activities^1^.

Traditionally, the surgeon performs genioplasty completely based on clinical experience and imaging data. Due to the commonly used oral incision approach, the surgical field is narrow and adjacent to important vessels and nerves. The bone cutting process is repeated for adjustments and can thus easily lead to complications, such as local asymmetry, bone fracture and nerve damage^2^.

Robot-assisted surgery is currently a research hotspot in the surgical field. The da Vinci system for robot-assisted endoscopic surgery has gradually shown operational stability in a large number of clinical experiments; this system can reduce the rates of intraoperative injury and bleeding, the occurrence of complications and the hospitalization time^3^. In the field of orthopedics, corresponding robot auxiliary systems have also begun to be clinically applied in hip arthroplasty^4^, spine surgery^5^ and other surgical areas. There is a certain degree of automation in grinding, which can improve the accuracy of surgery and reduce complications. Good results have been achieved in all aspects.

In the field of craniofacial surgery, most research teams are still in the stage of phantom surgery^6^, animal experiments and cadaver experiments. In 1998, Humboldt University reported the developed OTTO robot system as the first robot system applied in craniofacial surgery in the world. Using industrial robots as the platform and a Delta Kinematics robot as the form, a clinical trial of nail implantation for external ear reconstruction was conducted in 13 patients^7^. In 2001, the RobaCKa robot system developed at Karlsruhe University in Germany was used to drill and grind the skull surface automatically. However, the robot is large and has poor flexibility; the robot's only clinical application is skull grinding^8^. In summary, the above studies did not focus on applying facial contouring surgery, which has wide applications in craniofacial surgery. Moreover, some devices are still in the prototype stage^9–11^.

**Figure 1:**
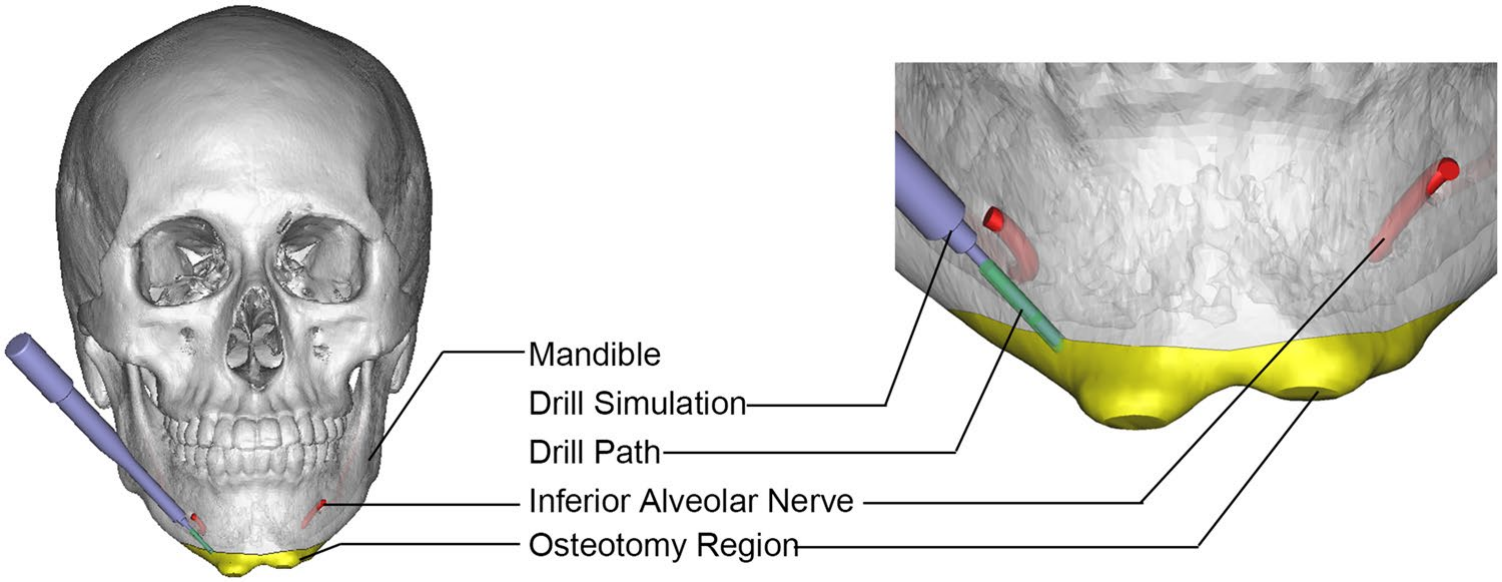
Mandibular deformity treated with genioplasty.

With the help of navigation technology, the surgeon can solve the problem of accurate surgical tool positioning. However, craniofacial contouring surgery involves high-stress conditions, such as those of drilling, grinding, osteotomy and other operations, which easily lead to a sense of fatigue and cannot guarantee the accuracy and stability of the operation in Fig. 1.

Currently, there are no commercial systems for robot-assisted craniofacial surgery. In previous studies, according to the characteristics of craniofacial surgery, our research team designed the CPSR-I surgical robot. We performed a series of surgical experiments using phantoms^12^ and animals^13,14^ and verified the related performance indicators of the mechanical structure, navigation system, control system and force feedback system.

In view of the problems in clinical operations, this research aimed to use the human–robot cooperation method in the operating room (OR) environment. Using navigation technology and force sensors, the surgeon obtained information for the judgment of robot control. The robot allowed accurate positioning and automatic drilling. This study focused on testing the clinical flow using the CPSR-I surgical robot in a real OR environment while accumulating experience and exploring and evaluating the efficacy of the robot clinically applied in genioplasty.

## Results

### Demographics data for patients

Among the 7 patients enrolled from July 2018 to July 2019, 1 patient failed to complete the follow-up and was excluded. A total of 6 patients (mean age 22.5 years) were followed up, including 5 female patients and 1 male patient. The demographic characteristics and postoperative assessment data of the participating patients are presented in Table 1. All patients were treated according to the described study protocol.

**Table 1:** Demographics, perioperative and postoperative assessment for patients.

No	1	2	3	4	5	6	Mean ± SD
**Demographics**							
Age (years)	18	21	18	22	30	26	22.50 ± 4.30
Sex^a^	M	F	F	F	F	F	–
Diagnoses^b^	1	2	1	2	2	1	–
**Perioperative and postoperative assessment**							
I.T. (min)	15	14	13	20	17	18	16.20 ± 2.40
S.T. (min)	80	100	90	150	180	70	111.67 ± 39.76
B.L. (ml)	80	120	100	200	200	60	126.67 ± 20.00
H.T. (days)	7	7	7	7	7	7	7
Complications^c^	None	None	None	None	None	None	–
A.E.	None	None	None	None	None	None	–
S.S.	None	None	None	None	None	None	–
D.E. (mm)^d^	2.29	2.93	2.05	2.46	1.35	2.07	2.19 ± 0.48
P.S.S. (1 month)	5	4	5	4	4	5	4.50
P.S.S. (6 months)	5	5	5	5	5	5	5

*I.T.* Installation time, *S.T.* Surgical time, *B.L.* Blood loss, *H.T.* Hospital time, *A.E.* Adverse events, *S.S.* Second surgery, *D.E.* Distance error, *P.S.S.* Patient’s satisfaction scale, *No.* Number, *S.D.* Standard deviation, *mm* millimeter, *min.* minutes, *ml.* milliliter.

^a^M = male, F = Female.

^b^1 = Chin asymmetry, 2 = Retrognathia.

^c^Complications are included with bone fracture, infection, nerve damage and local hematoma, all patients are followed for 6 months.

^d^Using the software interface, the designed preoperative mandibular data and the actual postoperative data were imported. The fitting algorithm of the software was used to register the two data sets. Then, the reference model was set as the postoperative skull data, while the test model was set as the preoperative skull data for the error analysis. Finally, the entry points of the hole paths were selected, and the corresponding precision error data were measured. These data were averaged over three times by one tester.

### Perioperative data for patients

The installation and operation of the entire robot system started synchronously. The total time required to install the navigation markers and debug the system was 16.2 ± 2.4 min. The entire operation lasted 111.67 ± 39.76 min. Intraoperative blood loss was 126.67 ± 20 ml. The patients were hospitalized for 7 days without complications or adverse events. Postoperative 3D CT was performed for the patients as performed preoperatively at 1 week after surgery. Preoperative and postoperative imaging data were obtained, and the distance error was 2.19 ± 0.48 mm (Table 1).

### Postoperative data for patients

No second surgery was required for any patient after the first treatment during the follow-up period. The patients were very satisfied with the results of the operation. One month after surgery, half of the patients were very satisfied, and the other patients reported a rating of 4 (4/5) for satisfaction. Six months after surgery, all the patients were very satisfied with the postoperative aesthetic results. All postoperative assessments of the surgical data of all 6 patients were recorded for analysis, as shown in Table 1.

### Error measurement of mechanical system

**Figure 2:**
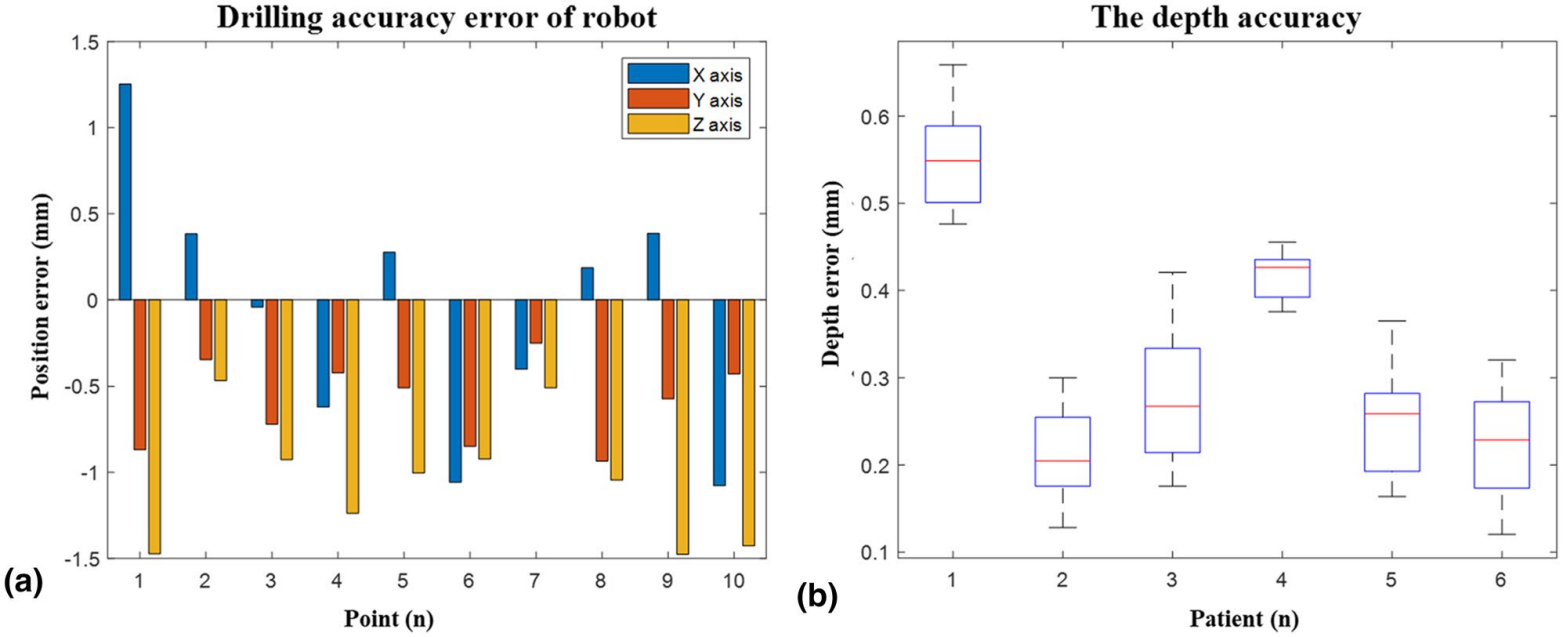
The accuracy of the robot system: (**a**) the drilling accuracy; (**b**) the depth accuracy.

During the process of the robot moving at a constant speed, the end position and posture *Po* of the robot under the optical navigation system are recorded. By using formula error =|[*Po*;1] − (*T* × [*Pd*;1])|, the drilling error (Fig. 2a) is obtained by comparing with the drilling path *Pd* designed before the operation (a total of 10 drilling paths for patient 4). Where *T* is the registration matrix between the robot coordinate system and the image coordinate system. The average drilling error is 1.38 ± 0.46 mm. Selecting the results of all patients, we obtain the depth error during the drilling process, as shown in Fig. 2b. The average depth error is 0.32 ± 0.14 mm.

### Force feedback data

**Figure 3:**
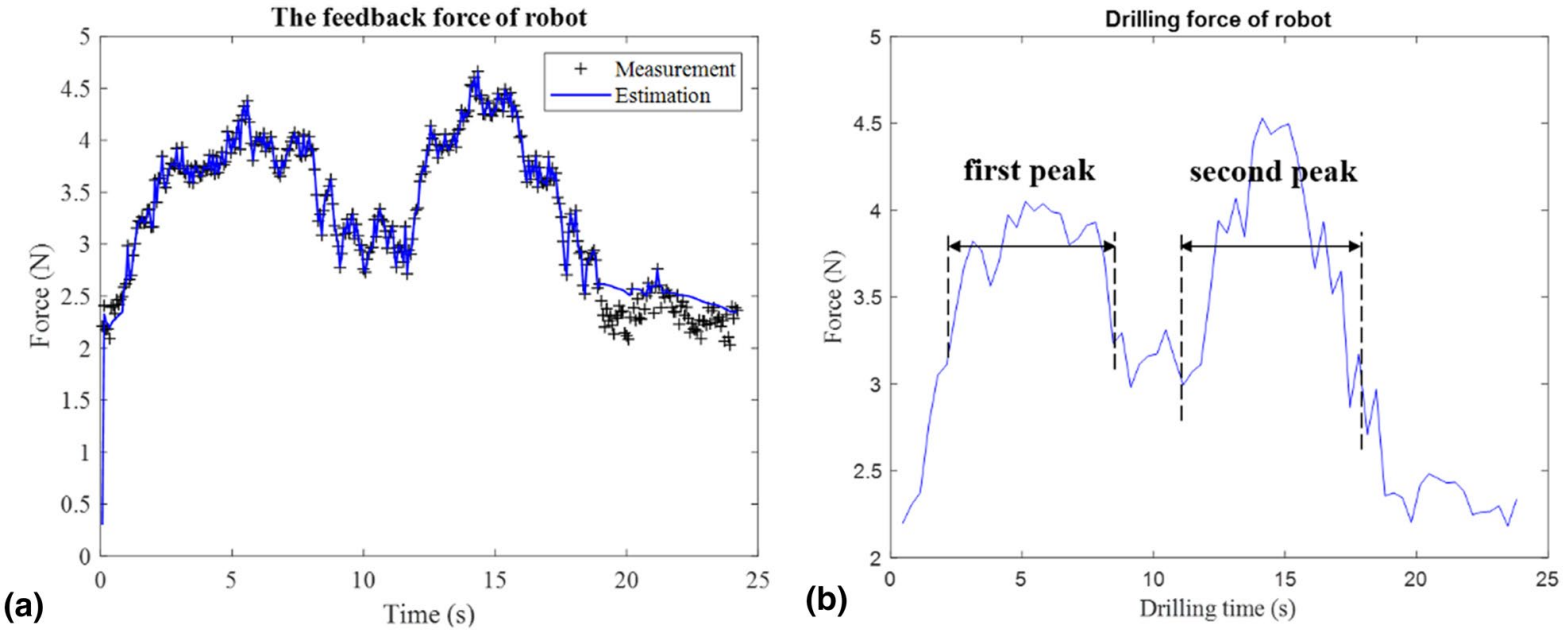
The drill force: (**a**) the feedback force by Kalman filter; (**b**) the force information during drilling.

The sensor performs high-frequency sampling, and the collected force feedback data will produce high-frequency, low-amplitude signal jitter, which will distort the useful signal during the transmission process, and it is difficult to resolve the useful force feedback signal. We employed a time-varying linear recursive Kalman filtering algorithm which combined past estimation errors and new measurement values to estimate future measurement values. Figure 3a shows the original force data signal and the filtered signal for patient 3. It is found that the use of Kalman filtering can filter out noise signals in real time based on dynamic information, thereby obtaining a good estimate of the true feedback force in the future.

By recording the force information, a curve of force versus time is obtained. In the force test results, all the groups are all M-shaped, and their graphic structures are similar to those in animal experiments. The experimental results of patient 3 obtained are shown in Fig. 3b. Due to the difference in the depth of the drilling site and the bone density, there are differences in the drilling time and the size of the drilling force, but they all show approximately the same trend of change.

## Discussion

The key steps of craniofacial surgery include drilling, grinding, cutting and other operations on bone tissue under great stress. To consider the special requirements of the patient's functional abilities and appearance, surgical incisions are generally designed to be hidden. In facial contouring surgery, an intraoral incision is mainly used, and the operating space is very limited due to the narrowness of the incision. Moreover, the incision is adjacent to an important neurovascular bundle; thus, the surgeon in charge of the surgery is under high pressure.

With the development of computer-aided surgery and surgical navigation technology, great progress has been made in preoperative surgical design and intraoperative navigation. However, under high-stress conditions, it is unavoidable for the surgeon to experience hand tremors. Shaking of the surgical instruments will affect the operational stability and accuracy and could lead to failure to meet the corresponding requirements of the preoperative design. Especially among novice surgeons, the lack of familiarity with and experience in operating surgical instruments can often lead to surgical complications.

Robot-assisted surgery has been a research hotspot in the medical field in recent years and has been widely used in eye surgery^15^, urological surgery^16^, general surgery^17^, neurosurgery^18^, orthopedic surgery^4^, ear, nose and throat (ENT) surgery^19^ and rehabilitation medicine^20^. The advantage is that it can reduce the tremor of human hands, thus preventing operation errors caused by fatigue and increasing the positional accuracy of the operation. Currently, surgical robot systems are widely used mainly in endoscopic surgery. In the field of plastic surgery, they are mainly applied in microsurgery^21^, transoral repair surgery^22^, breast reconstruction surgery^23^ and flap surgery^24^. Due to the high cost of general-purpose medical robots, all surgical departments are actively carrying out experiments to promote the application of small specialized medical robots.

In current international medical equipment standards, risk control is an important factor in the design stage. Both ISO-14971^25^ and IEC-60601-77^26^ have corresponding provisions for medical electrical equipment. Due to current issues related to medical ethics, robots cannot have complete automatic functions^27^. Therefore, the development of systems for robot-assisted surgery should focus on researching human–robot interactions, cooperation and control. For some simple repetitive operations or operations with high precision requirements, the robot can perform procedures with more intelligence under the supervision of the surgeon. However, the final decision and control should be in the hands of the surgeon.

The main challenge of robotic surgery lies in the safety of surgery, the naturalness of interaction, and accuracy, which greatly limit the development of surgical robots^28^. Aiming at the characteristics of mandibular osteotomy, which has a narrow operating space and almost blind vision, augmented reality navigation is used for visual guidance to achieve precise positioning of the robot.

In terms of the safety of the operation, drilling, grinding, osteotomy and other operations are carried out under high stress conditions. In addition, to the restrictions of the operation conditions, surgeon is prone to fatigue and unstable to hold the reciprocating saw. This may result the damage of nerve and blood vessels. In this process, the accuracy and stability of real-time operation cannot be guaranteed, which increase the complications of surgery. We adopt the method of robot-assisted surgery, and adopt the automatic drill bone control strategy combining position and force conditions, and design an adaptive fuzzy controller to improve the safety and stability of the operation.

During the process of the robot moving at a constant speed, the end position and posture *Po* of the robot under the optical navigation system are recorded. By using formula error =|[*Po* ;1] − (*T* × [*Pd*; 1])|, the drilling error is obtained by comparing with the drilling path *Pd* designed before the operation (a total of 10 drilling paths for patient 4). Where *T* is the registration matrix between the robot coordinate system and the image coordinate system. Selecting the results of all patients, we obtain the depth error during the drilling process, as shown in Fig. 2b. The CT measurements contain the surgeon's operating errors, which are greater than those of the robotic system.

The results of this study show that robot-assisted surgery is a good method based on human–robot cooperation to guide accurate osteotomy lines through drill holes. In terms of accuracy, the osteotomy and grinding performed after the creation of the guide hole increased the measurement error. However, the final experimental results were still within an acceptable range. In addition, the operation time and blood loss showed that the application of robot-assisted surgery had no obvious impact on the patient and did not carry additional risk. The advantage of the system is that as long as the surgeon places the robotic arm at the corresponding drilling position, according to the guidance of the navigation system, the robot can automatically drill and retreat, reducing the workload of the surgeon. After the drilling process, use of the actual osteotomy line according to the preoperative drilling plan is conducive to surgical guidance.

This study also has some deficiencies. First, the visual guidance of the navigation system based on augmented reality is still insufficient, and further interactive assistance is needed to solve the problem of depth perception for judgment. Second, the original design of the mechanical structure of the CPSR-I is relatively heavy and inconvenient. The end-effector sometimes blocked the surgeon’s field of view and interfered with the space of other medical equipment. What’s more, other craniofacial surgical procedures are also needed to be evaluated in the following experiments. These problems need to be further optimized in later versions of the CPSR-I robot.

## Conclusions

This is the first report of robot-assisted mandibular contouring surgery in clinical practice. Based on the early results of the 6 patients, the robot-assisted approach appears to be a safe and effective strategy for genioplasty. Further clinical evidence and more cases involving this technique are required.

## Methods

### Structure of robot system

**Figure 4:**
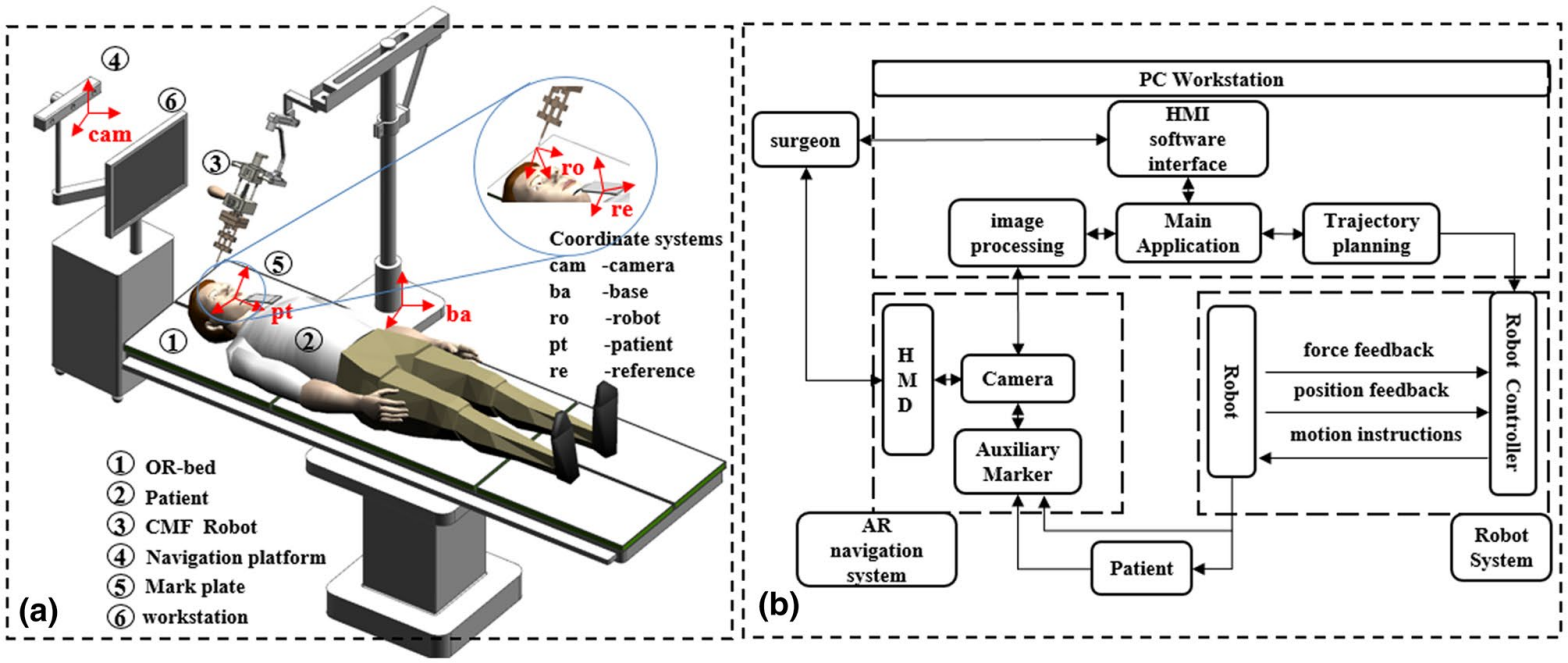
The robot system: (**a**) the structure diagram; (**b**) the system structure frame.

The robot-assisted craniomaxillofacial surgery system developed by Shanghai Jiao Tong University, which can accurately locate in a narrow operating space and improve the success rate of surgery (Fig. 4). Real-time monitoring of intraoperative bone strength through force feedback is achieved. The aluminum alloy material is used, which reduces the weight of the mechanism while ensuring strength and rigidity. At the same time, it is equipped with a gravity compensation mechanism to reduce the position accuracy error caused by gravity. Besides, the occlusal splint is combined to assist in precise positioning. The system mainly consists of a robot subsystem and an augmented reality navigation subsystem.

### Key features of the robotic assistance device

**Figure 5:**
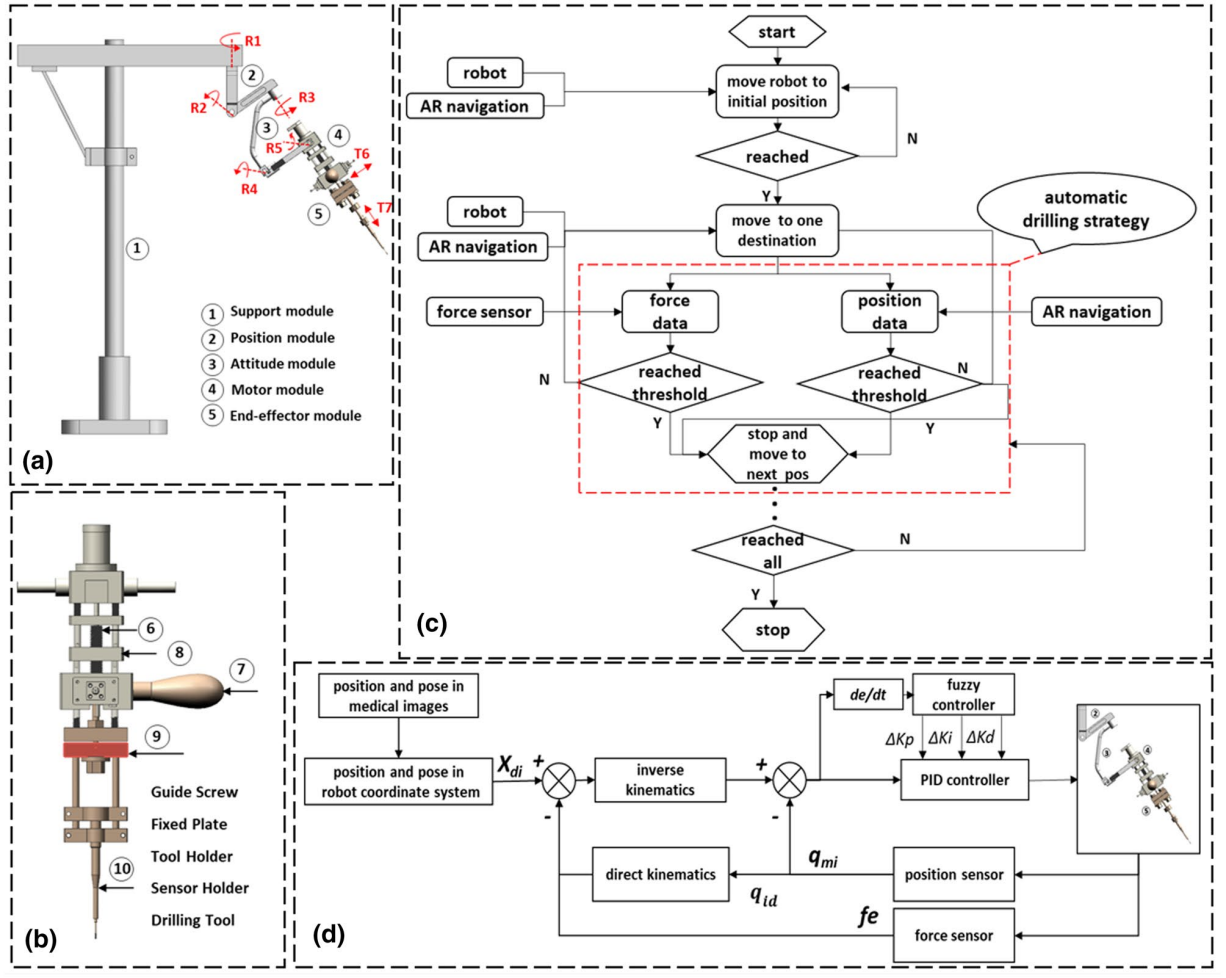
The robot system: (**a**) the structure frame diagram; (**b**) the structure of the force feedback system; (**c**) the automatic drilling strategy diagram; (**d**) the structure of the control system.

Among them, the robot subsystem is mainly composed of a 7 degree-of-freedom (DOF) robot (support, position, attitude, power and mechanical surgical instrument), force feedback module and fuzzy control module, which assists the surgeon in completing the operation. The structural design of position and posture decoupling reduces the difficulty of control, Suitable for robot operation in narrow space with craniofacial contouring surgery. The augmented reality navigation subsystem is composed of a marker complex, optical camera, perspective helmet, and navigation software, among other components. It is used for preoperative planning and intraoperative guidance of the robot’s position (Fig. 5).

### The force feedback function

In robot-assisted surgery, effective force perception cannot be achieved because the system has no direct contact with tissue. Therefore, we considered designing a force feedback subsystem (Fig. 5b). Force feedback during the manipulation of surgical instruments can help the surgeon perform the operation better.

The force sensor (JHBM-M, Jin Nuo, China) is integrated into the surgical instrument module to detect force during drilling. The sensitivity, accuracy and measurement error are 1.5 ± 0.2 mv/V, 0.5% F S and 0.5% F S, respectively. The data acquisition card (USB2815, ATR) collects force signals at a sampling frequency of 250 kHz. Since there will be some noise signals during the drilling process, a filter should be designed to filter out jitter signals. To avoid external interference, a Kalman filter is adopted to improve the accuracy of the force data. The principle is as follows:

1 $${\widehat{x}}_{\stackrel{-}{k}} =A*{\widehat{x}}_{k-1}+B*{u}_{k-1}$$

2 $${P}_{\stackrel{-}{k}}=A*{P}_{k-1}*{A}^{T}+Q$$

3 $${K}_{k}=\frac{{P}_{\stackrel{-}{k}}*{H}^{T}}{H*{P}_{\stackrel{-}{k}}*{H}^{T}+R}$$

4 $${\widehat{x}}_{k}={\widehat{x}}_{\stackrel{-}{k}}+{K}_{k}*({z}_{k}H*{\widehat{x}}_{\stackrel{-}{k}})$$

5 $${P}_{k}=(I-{K}_{k}*H)*{P}_{\stackrel{-}{k}}$$

${\widehat{x}}_{k-1}$ and ${\widehat{x}}_{k}$ represent the posterior state estimates at *k-*1 and *k* times, respectively, and ${\widehat{x}}_{\stackrel{-}{k}}$ is the prior state estimate at *k* times. ${P}_{k-1}$ and ${P}_{k}$ represent the posterior covariance at *k*-1 and *k* times, respectively. ${P}_{\stackrel{-}{k}}$ is a prior estimate covariance of *k* time. *H* is the transformation matrix from state variables to measurements. ${z}_{k}$ is the measured value, ${K}_{k}$ is the filter gain matrix, and *A* is the state transition matrix.

### Automatic drilling control strategy

To compensate for the lack of depth information, the force sensor is used to monitor the feedback force during drilling in real time. Due to the uneven bone density distribution, the simple force feedback principle was used to determine whether the two bone density layers will cause greater errors in bone penetration, leading to inaccurate and unstable drilling (Fig. 5c).

Although the structure of the human body is different from that of beagle dogs, the trend of force change in animal experiments is still instructive for clinical experiments. Therefore, we consider the combination of the force feedback principle and position control principle, adopting the force-first approach, and improving the safety and accuracy of the operation by setting the threshold range of the task space for safe robot operation and obtaining feedback during the drilling process. When the position of the robot exceeds the set threshold, the robot will stop automatically to avoid damaging the maxillofacial tissue. At the same time, when the drilling force decreases sharply for the second time and reaches the force threshold, it is considered that the bone has been drilled through and the robot stops moving.

### Design of the control system

As the execution unit of surgery, the stability and reliability of the robot is the key to precise surgery. Considering the complex and changeable operating environment, the control system has strong robustness and adaptability to the environment.

Since the robot is a non-linear multiple-input multiple-output system stable and accurate operation is the key to the surgical task. Due to the influence of uncertain factors such as external interference and complicated external environment, it is difficult to establish an accurate mathematical model. Conventional proportional integral derivative (PID) control^29,30^ is not suitable for surgical robot systems with a changeable environment because of its parameters cannot be adjusted in real time, while fuzzy control^31–33^ does not require accurate mathematical models of the controlled object.

Therefore, we designed an adaptive fuzzy controller^34–36^ that combines PID control and fuzzy control to be used in the motion control of the CPSR-I surgical robot to achieve precise control of the surgical robot. An adaptive fuzzy system is designed (Fig. 5d).

The two input variables of the fuzzy controller are the error e and the error rate ${e}_{c}$; the three output variables are the proportional coefficient ${K}_{p}$, the integral coefficient ${K}_{i}$ and the differential coefficient ${K}_{d}$. In the control process, the fuzzy controller adjusts the coefficients continuously by the error e and error rate ${e}_{c}$ to achieve fast and stable control. $e$ is the position error of the joint,$e\in [-\mathrm{3,3}]$, ${e}_{c}\in [-\mathrm{3,3}]$, $\Delta {K}_{p}\in [-\mathrm{5,5}]$, $\Delta {K}_{i}\in [-\mathrm{5,5}]$, $\Delta {K}_{d}\in [-\mathrm{5,5}]$.

The linear relationship of the conversion formula is:

6 $${y}_{0}={k}_{x}\left({x}_{0}-\frac{{x}_{max}+{x}_{min}}{2}\right)+\frac{{y}_{max}+{y}_{min}}{2}$$

7 $${k}_{x}=\frac{{y}_{max}-{y}_{min}}{{x}_{max}-{x}_{min}}$$

Among them, $[ {x}_{max} {x}_{min}]$ is the variable interval of the standard membership function and $[ {y}_{max} {y}_{min}]$ is the actual variable interval of the standard membership function.

In the process of defuzzification, the gravity method is used for defuzzification according to the rule of Mamdani maximum-minimum gravity method. The expression is:

8 $$u_{{K_{m} }} = \mathop \sum \limits_{i = 1}^{n} K_{mi} u\left( {\Delta K_{mi} } \right)/\mathop \sum \limits_{i = 1}^{n} u_{i} \;\;\;\;\;\;\;m = p, \, i, \, d$$

Among them, ${u}_{{K}_{m}}$ is the result of defuzzification, and ${u}_{i}$ is the membership function.

### Augmented reality navigation system

The augmented reality navigation system adopted in this study is mainly composed of a mark complex, optical camera, perspective helmet and navigation software. A preliminary basic study^37^ and clinical studies^38–41^ were conducted to verify the reliability of the system. The corresponding mark plate is located below the patient in the supine position, 30 degrees from the sagittal position of the head. Navigation tracking is carried out using an optical camera (Micron Tracker, Claron, Canada) in the actual operating environment. 3D printing (Projet 660pro, 3D Systems, USA) was applied to obtain a personalized navigation mark complex, which was preoperatively sterilized. The intraoperative registration system tracks the geometric center of the corresponding marker complex via the optical camera. Using the automatic registration program, the workstation performs calculations and tracking in real time. Then, the surgeon performs the surgery using real-time visual navigation.

### Patient selection

Based on previous experiments, a preliminary clinical study was conducted to verify the efficacy and safety of the system. The patients in this study were recruited from the Shanghai 9th People's Hospital, School of Medicine, Shanghai Jiao Tong University. The inclusion criteria were as follows: (1) indication for genioplasty; (2) age of 18–30 years; (3) male or female gender; and (4) provision of informed consent. The exclusion criteria were as follows: (1) complicated cosmetic surgery; (2) serious systemic diseases; (3) no informed consent; (4) an inability to accept treatments; and (5) any indication requiring removal from the study. This study was approved by the independent ethics committee of Shanghai Ninth People’s Hospital affiliated with Shanghai Jiao Tong University, School of Medicine (No. 2017-319-T239). The clinical study was conducted according to the requirements of the ethics committee.

### System installation

The chief surgeon was a senior craniofacial specialist (more than 15 years of clinical experience, with an annual average of more than 150 craniofacial operations). Before the operation, the team of engineers trained the surgeons, anesthetist and nurses on overall system use to ensure that the operation team could clearly understand the whole process of robot-assisted surgery and avoid adverse events. In addition, the engineering team installed the system in the OR, which required approximately 15 min. Furthermore, the team monitored the operation of the system to prevent accidents.

### Intraoperative execution

During the operation, after the surgeon opened the incision, the soft tissue was fully dissected, and the osteotomy area in need of treatment was exposed. After full stripping, the marker complex was fixed to the corresponding chin area, and the optical camera was adjusted for tracking and navigation. Finally, the drilling module system was assembled and installed on the robot arm.

With the help of the navigation system, the surgeon held the surgical instrument to the corresponding surgical drilling position. After the surgeon confirmed the position of the drill hole of the end tool, the robot auxiliary device was self-locked, and the motion of the auxiliary system was disabled, thus improving the positional accuracy of the operation. For the sake of safety, a foot switch was available for cutting off movement of the surgical instrument module and avoiding injury to the patient.

During the drilling process, the corresponding control changes were made according to the information provided by the navigation and force feedback systems. When the corresponding threshold value was exceeded, the mechanical arm automatically stopped and automatically returned to a safe position to prevent damaging soft tissue. Moreover, the surgeon could execute an emergency stop of the robot system at any time throughout the process. After completion of the drilling process, the surgeon used a reciprocating saw for the osteotomy according to the guided osteotomy curve and ground the corresponding edges and corners.

### Postoperative assessments

**Figure 6:**
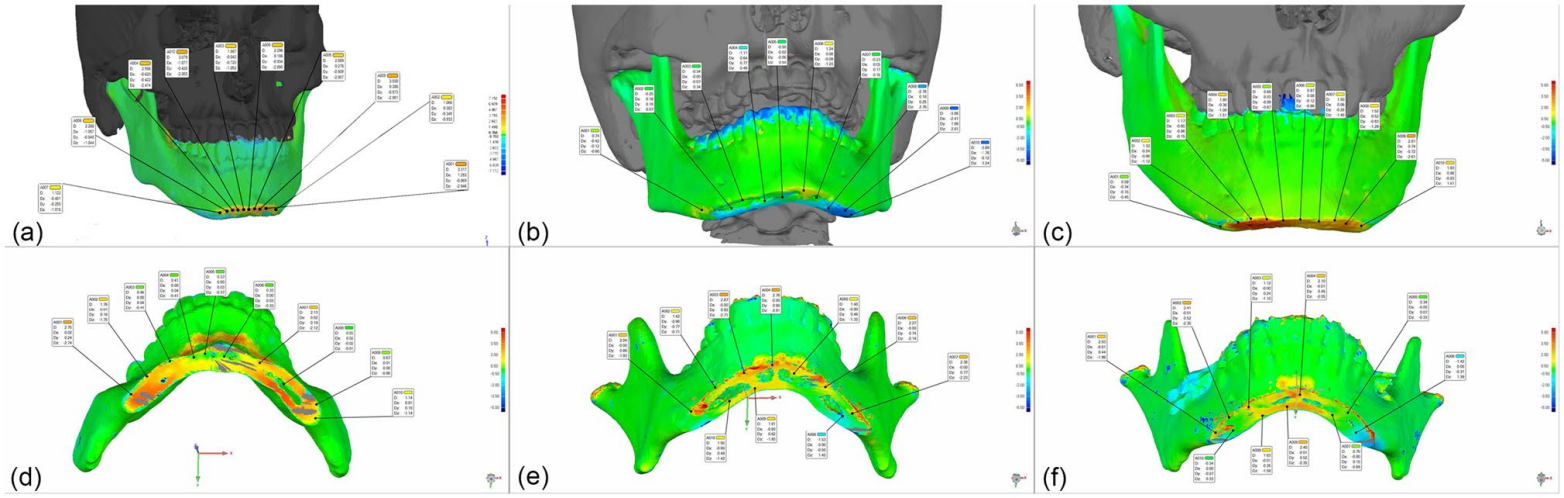
Accuracy analysis of genioplasty for all patients.

Postoperative assessments were scheduled for 1 week, 1 month, and 6 months postoperatively. Postoperative 3D CT was performed for each patient as performed preoperatively at 1 week after surgery, and the data were saved in DICOM format. In the 3D reconstruction software (Mimics 21, Materialise, Belgium), the postoperative mandible of the patient was reconstructed using the same method and saved in STL format. In fitting 3D imaging software (Geomagic Control 2015, 3D Systems, USA), the preoperative osteotomy plane was compared with the actual osteotomy data after the surgery. Since the studied patients mainly underwent changes to mandibular structures during the surgery, the original drilling start points were selected as points for the error analysis (Fig. 6).

The data from three measurements by one tester were averaged. Then, a 5-point Likert scale^42^ (1 indicating very dissatisfied, 5 indicating very satisfied) was used to assess the patients' satisfaction with the postoperative aesthetic results at 1 month and 6 months after surgery. Surgical data, adverse surgical events, and complications were recorded.

### Ethical approval

All procedures performed in studies involving human participants were in accordance with the ethical standards of the institutional and/or national research committee and with the 1964 Helsinki declaration and its later amendments or comparable ethical standards. This study was approved by the independent ethics committee of Shanghai Ninth People’s Hospital affiliated with Shanghai Jiao Tong University, School of Medicine (No. 2017-319-T239).

### Informed consent

Informed consent was obtained from all individual participants included in the study.

## Data Availability

Correspondence and requests for materials and data should be addressed to G.C.
